# Impact on pregnancy outcomes of intermittent preventive treatment with sulfadoxine-pyrimethamine in urban and peri-urban Papua New Guinea: a retrospective cohort study

**DOI:** 10.1186/s12936-024-05010-0

**Published:** 2024-07-05

**Authors:** Philip Cellich, Holger W. Unger, Stephen J. Rogerson, Glen D. L. Mola

**Affiliations:** 1 Division of Obstetrics and Gynaecology, School of Medicine and Health Sciences, Port Moresby General Hospital, University of Papua New Guinea, Port Moresby, Papua New Guinea; 2 Department of Obstetrics and Gynaecology, Canterbury Hospital, 575 Canterbury Road, Campsie 2194, NSW Australia; 3 Menzies School of Health Research, Charles Darwin University, PO Box 41096, Casuarina 0810, NT Australia; 4 Department of Obstetrics and Gynaecology, Royal Darwin Hospital, Darwin, NT Australia; 5 Department of Clinical Sciences, Liverpool School of Tropical Medicine, Liverpool, UK; 6 Department of Infectious Diseases, University of Melbourne, The Doherty Institute, Melbourne, VIC Australia; 7 Department of Medicine, University of Melbourne, The Doherty Institute, Melbourne, VIC Australia; 8 School of Medicine and Health Sciences, University of Papua New Guinea, Port Moresby, Papua New Guinea

**Keywords:** *Plasmodium falciparum*, Malaria, Intermittent preventive treatment, Pregnancy outcome, Low birth weight, Anaemia, Western Pacific

## Abstract

**Background:**

Intermittent preventive treatment in pregnancy with sulfadoxine-pyrimethamine (IPTp-SP) reduces malaria-attributable adverse pregnancy outcomes and may also prevent low birth weight (< 2,500 g) through mechanisms independent of malaria. Malaria transmission in Papua New Guinea (PNG) is highly heterogeneous. The impact of IPTp-SP on adverse birth outcomes in settings with little or no malaria transmission, such as PNG’s capital city Port Moresby, is unknown.

**Methods:**

A retrospective cohort study was conducted amongst HIV-negative women with a singleton pregnancy who delivered at Port Moresby General Hospital between 18 July and 21 August 2022. The impact of IPTp-SP doses on adverse birth outcomes and anaemia was assessed using logistic and linear regression models, as appropriate.

**Results:**

Of 1,140 eligible women amongst 1,228 consecutive births, 1,110 had a live birth with a documented birth weight. A total of 156 women (13.7%) did not receive any IPTp-SP, 347 women (30.4%) received one, 333 (29.2%) received two, and 304 (26.7%) received the recommended ≥ 3 doses of IPTp-SP. A total of 65 of 1,110 liveborn babies (5.9%) had low birth weight and there were 34 perinatal deaths (3.0%). Anaemia (haemoglobin < 100 g/L) was observed in 30.6% (243/793) of women, and 14 (1.2%) had clinical malaria in pregnancy.

Compared to women receiving 0–1 dose of IPTp-SP, women receiving ≥ 2 doses had lower odds of LBW (adjusted odds ratio [aOR] 0.50; 95% confidence interval [CI] 0.26, 0.96), preterm birth (aOR 0.58; 95% CI 0.32, 1.04), perinatal death (aOR 0.49; 95% CI 0.18, 1.38), LBW/perinatal death (aOR 0.55; 95% CI 0.27, 1.12), and anaemia (OR 0.50; 95% CI 0.36, 0.69). Women who received 2 doses versus 0–1 had 45% lower odds of LBW (aOR 0.55, 95% CI 0.27, 1.10), and a 16% further (total 61%) reduction with ≥ 3 doses (aOR 0.39, 95% CI 0.14, 1.05). Birth weights for women who received 2 or ≥ 3 doses versus 0–1 were 81 g (95% CI −3, 166) higher, and 151 g (58, 246) higher, respectively.

**Conclusions:**

Provision of IPTp-SP in a low malaria-transmission setting in PNG appears to translate into substantial health benefits, in a dose–response manner, supporting the strengthening IPTp-SP uptake across all transmission settings in PNG.

**Supplementary Information:**

The online version contains supplementary material available at 10.1186/s12936-024-05010-0.

## Background

Malaria in pregnancy has egregious consequences for maternal, fetal, and infant health [1]. Malaria in pregnancy is a cause of anaemia, impacting on maternal well-being and worsening the risks and outcomes of postpartum haemorrhage [2], and continues to be a cause of maternal death in endemic settings [3]. *Plasmodium falciparum*-infected red bloods cells sequester in the placental intervillous space. Downstream effects of placental sequestration, include fetal growth restriction, preterm delivery, fetal loss and lower birth weight [4]. Placental malaria has been associated with malaria risk and longer term adverse health outcomes in surviving children [5]. *Plasmodium vivax*, the geographically most widely distributed cause of malaria, has also been implicated in adverse maternal and fetal outcomes [6].

The World Health Organization (WHO) recommends a three-pronged approach to reduce the adverse impacts of malaria in pregnancy. This includes prompt detection and treatment of clinical malaria; the use of insecticide-treated bed nets; and monthly intermittent prevention treatment in pregnancy with sulfadoxine-pyrimethamine (IPTp-SP) from second trimester until delivery [7]. IPTp-SP is endorsed by the WHO for malaria-endemic countries in sub-Saharan Africa only. The purpose of IPTp-SP is to provide presumptive treatment of malaria infection (including placental infection) and a degree of chemoprophylaxis to prevent new infection. Most women with infection are asymptomatic and many carry low density infections [8]. Currently available point-of-care tests such as microscopy and rapid diagnostic tests have poor sensitivity and miss at least half of women with placental malaria, making IPTp an indispensable strategy [8, 9]. IPTp-SP has been shown to substantially reduce low birth weight (LBW; < 2,500 g), a predictor of survival and adverse child health outcomes [3].

There is emerging evidence of possible non-malarial benefits of IPTp-SP for maternal and fetal health [10–12]. IPTp-SP may have activity against sexually transmitted infections such as chlamydia and gonorrhoea, which are associated with preterm birth [10]. Furthermore, IPTp-SP has been shown to reduce LBW amongst women without malaria or sexually transmitted infections [10]. IPTp-SP has been associated with improved gestational weight gain, a predictor of birthweight [13, 14]. IPTp-SP may alter maternal carriage of enteric pathogens associated with lower gestational weight gain and appears to have a direct therapeutic effect on nutritional deficiency-induced enteric dysfunction [15, 16]. In clinical trials comparing IPTp-SP with IPTp using dihydroartemisinin-piperaquine, babies of women randomized to IPTp-SP were larger despite superior anti-malarial efficacy of dihydroartemisinin-piperaquine [11].

Papua New Guinea (PNG), a country of approximately 12 million inhabitants located in the Western Pacific region, has moderate-to-high transmission of both *P. falciparum* and *P. vivax*. There is a high burden of LBW, perinatal death, other adverse pregnancy outcomes and maternal and child health indicators in PNG [17]. In 2009, PNG became the only country outside of sub-Saharan Africa to adopt IPTp-SP. PNG’s National Malaria Strategic Plan 2014–2018 recommends administration of three doses of IPTp-SP, without reference to local transmission intensity [18]. It is unclear whether there is a benefit of IPTp-SP in urbanized areas of PNG, where malaria transmission and malaria-attributable adverse birth outcomes are likely to be low or absent. Considering the possible non-malarial benefits of IPTp-SP, it was hypothesized that IPTp-SP retains a substantial benefit for the prevention of LBW and other adverse birth outcomes in low transmission areas of PNG. A study was designed to evaluate the impact of IPTp-SP on LBW and other pregnancy outcomes at Port Moresby General Hospital in PNG’s capital city, Port Moresby.

## Methods

### Aim

The aim of this study was to determine the impact of IPTp-SP on adverse pregnancy outcomes in a low-transmission setting of PNG.

### Study design

A retrospective cohort study was conducted amongst women who had a registered birth at Port Moresby General Hospital, Port Moresby, PNG.

### Setting

The study was conducted in the maternity wards of Port Moresby General Hospital in PNG. Port Moresby General Hospital provides maternity care for pregnant women residing in PNG’s National Capital District and surrounding areas. Between 1979 and 2015, annual hospital reports show that 88–96% of birthing women at Port Moresby General Hospital had booked at the capital city antenatal clinics. About 10–15% of women who attend Port Moresby antenatal clinics reside outside the National Capital District, in the adjacent Central Province. Malaria transmission is seasonal on the south coast of PNG [19]. Data from the 2019/20 PNG Malaria Indicator Survey suggest that malaria transmission in the National Capital District is low or absent. Of individuals screened for malaria infection, 0.0% and 0.2% (all *P. vivax*) tested positive by rapid diagnostic test and light microscopy, respectively [20]. In Central Province, 0.8% and 1.3% of screened individuals were positive by rapid diagnostic test and light microscopy, respectively [20].

### Participants and data collection

Participants included HIV-negative women with a singleton pregnancy who had a registered birth at Port Moresby General Hospital during the period 18 July 2022 to 21 August 2022, and who had data available on IPTp-SP use during pregnancy. The hospital birth register was used to identify women.

### Variable definitions

Participants were grouped by receipt of IPTp-SP, categorized as 0–1 dose of IPTp-SP versus 2 or more doses of IPTp-SP. To evaluate dose–response relationships, a second IPTp-SP exposure variable was coded: 0–1 dose, 2 doses, and 3 or more doses of IPTp-SP. These groupings were chosen based on observations from other settings demonstrating impacts on pregnancy outcomes amongst women receiving at least two doses, with minimal impacts noticed amongst women receiving one compared to no dose [10]. Three or more doses reflects PNG policy and was previously shown to be superior to two doses in high transmission settings in sub-Saharan Africa [21]. Key potential confounders that were considered included gravidity (categorized as primigravid/secundigravid versus multigravida), region of origin (Highlands origin versus others), current residence (peri-urban/rural versus urban), number of antenatal visits (0–3 versus 4 or more), and educational status (no schooling to grade 7 versus grade 8 and above).

LBW (< 2500 g) was the primary outcome measure. Stillbirths were defined as birth of a fetus without signs of life ≥ 22 gestational weeks, or when gestational age was unavailable, with a birth weight of ≥ 500 g. Perinatal death was defined as stillbirths and early neonatal deaths (neonatal deaths < 7 days of age). Gestational age was based on the treating clinician’s synthesis of all available information relating to last menstrual period, quickening, fundal height at booking, ultrasound findings and, occasionally, application of a Dubowitz score. Adverse birth outcome was defined as a composite of LBW and perinatal death. Routine testing for malaria parasitaemia was not performed, but symptomatic women had light microscopy and/or rapid diagnostic testing performed on peripheral blood. Anaemia was defined as a haemoglobin measurement < 100 g/L at delivery, or in late pregnancy when a delivery haemoglobin measurement was not taken.

### Data sources/management

Birth register data, hospital medical records, and antenatal clinic books were reviewed. Relevant data were extracted using a data collection proforma, including documented number of SP doses, maternal sociodemographic characteristics, obstetric history, haemoglobin, birth weight and other birth outcomes. Measurement of haemoglobin was predominantly by laboratory assay, with some peripheral clinics using point of care testing. Most birth weight measurements were taken by health care workers on the labour ward using mechanical scales, while a small number were taken in the special care nursery with electronic scales. Data were entered and stored in a password-protected database.

A minimum of 1,110 eligible women with a singleton live birth were needed to demonstrate a reduction in the odds of LBW by 40% from 17.0 to 10.2% in women receiving ≥ 2 doses of SP compared to women receiving 0–1 dose with 80% power and a two-sided alpha of 0.05. These estimates were informed by findings of an earlier clinical trial of IPTp in PNG [14]. To accommodate for fetal loss, multiple gestation, HIV infection, and missing data (SP exposure), medical records of a total of 1,228 women were reviewed (~ 10% additional recruitment). A subset of women (every tenth woman in the birth register) was invited to participate in a brief structured questionnaire survey evaluating the ownership and use of insecticide-treated bed nets, characteristics of administration of IPTp-SP, and self-reported side effects that women attributed to IPTp-SP.

### Statistical methods

Data were presented as % (number/total) for categorical variables, and as mean (standard deviation, SD) for continuous parametric variables. Basic comparative statistics included the Chi-squared tests for categorical variables, the unpaired t-test for continuous parametric variables, and logistic regression to calculate crude odds ratios. Maternal age, gravidity, highlands origin, number of antenatal visits, education status, maternal stunting, body mass index, iron supplementation, bed net use, and newborn sex were assessed as potential confounders, based on previous findings from PNG and elsewhere [14, 22]. Variables were considered confounders if they altered the odds ratio (OR) of LBW or anaemia in relation to IPTp exposure by ≥ 10% (Supplemental Tables 1 & 2). Confounders were included in multivariable logistic or linear regression models, as applicable. The possibility of effect modification of the relationship between IPTp exposure and LBW by gravidity and number of antenatal visits was evaluated by fitting an interaction term. Presence of collinearity between number of antenatal visits and number of SP doses was ruled out using collinearity diagnostics (*collin* function in Stata, variance inflation factor < 5 was accepted as absence of significant collinearity).

## Results

**Fig. 1:**
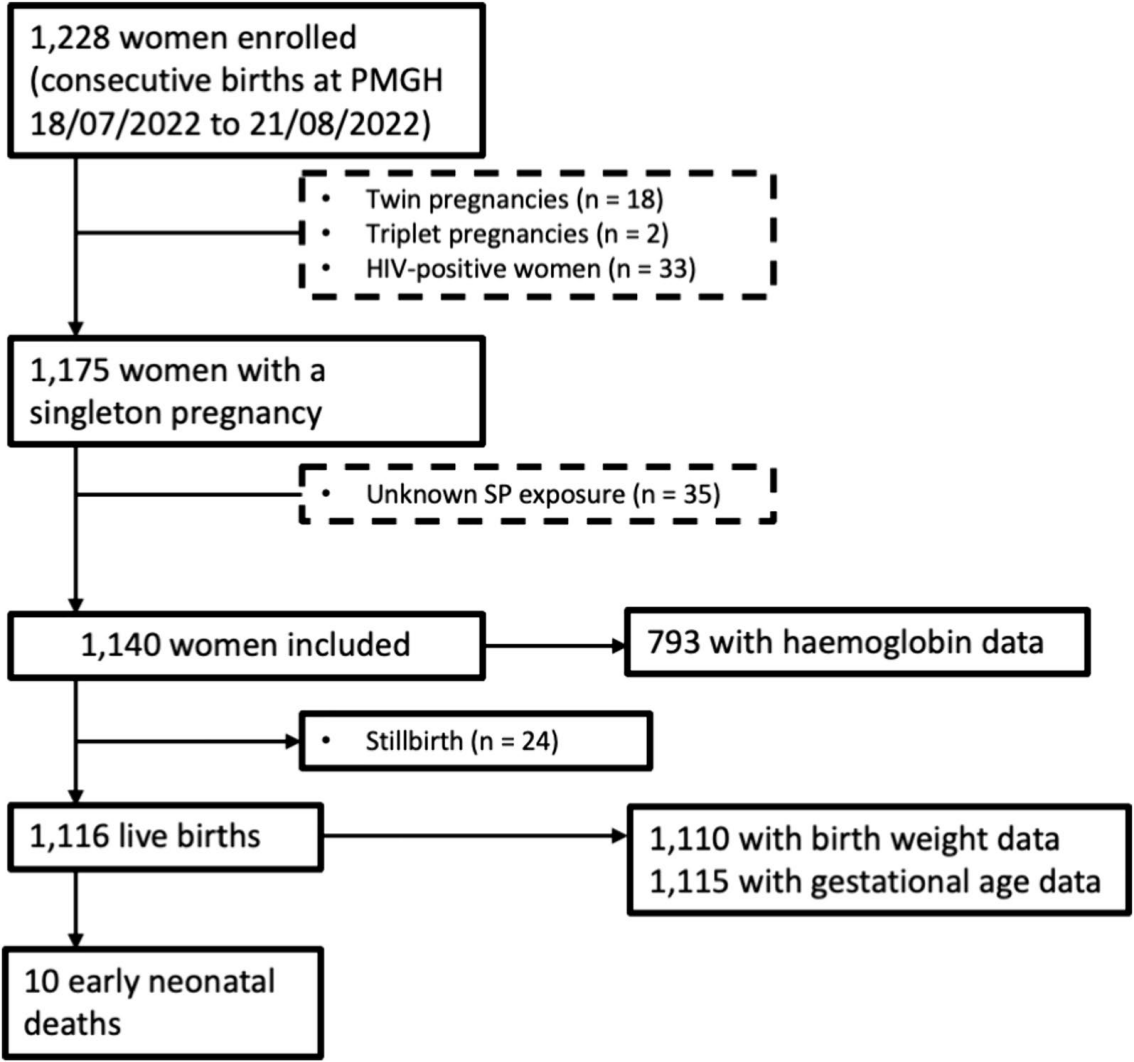
Participant flowchart

Of 1,228 women who delivered at Port Moresby General Hospital during the study period, 1,140 were considered in the analysis and 1,110 women had a live birth with a documented birth weight (Fig. 1).

Sociodemographic and clinical characteristics of study participants are summarized in Table 1. Nearly 40% of women were primigravidae, and most had ancestry in the Highlands or Southern regions of PNG. Approximately half resided in urban areas, and 113 women (9.9%) who had come to deliver at Port Moresby General Hospital had their residence in a rural location outside of Port Moresby. Ninety percent of women had at least one antenatal visit but only one-fifth of women had the recommended eight antenatal visits. Amongst participants, 94% of women had a vaginal birth.

**Table 1:** Characteristics of women at antenatal enrolment, overall and by receipt of sulfadoxine-pyrimethamine (SP)

	All women (n = 1,140)		SP 0–1 doses (n = 503)		SP ≥ 2 doses (n = 637)	
Age, in years	26.6	(6.0)	26.1	(6.3)	27.0	(5.8)
Gravidity						
Primigravid	38.4	(439)	39.4	(198)	37.8	(241)
Secundigravid	28.5	(324)	27.8	(140)	28.9	(184)
Multigravid	33.2	(377)	32.8	(165)	33.3	(212)
Region of Origin						
Southern	47.5	(542)	52.7	(265)	43.5	(277)
Highlands	40.4	(461)	36.4	(183)	43.6	(278)
Islands	4.7	(53)	4.4	(22)	4.9	(31)
Momase	7.4	(84)	6.6	(33)	8.0	(51)
Place of Residence						
Urban	47.9	(546)	39.4	(198)	54.6	(348)
Settlement	30.3	(345)	34.0	(171)	27.3	(174)
Peri-urban/Rural	21.8	(249)	26.6	(134)	18.1	(115)
Antenatal clinic booking						
No	7.8	(89)	17.7	(89)	0.0	(0)
Yes	92.2	(1,051)	82.3	(414)	100.0	(637)
Number of antenatal clinic visits						
None	7.8	(89)	17.7	(89)	0.0	(0)
1–3	31.0	(353)	51.7	(260)	14.6	(93)
4–7	44.7	(509)	26.6	(134)	58.9	(375)
8 +	16.6	(189)	4.0	(20)	26.5	(169)
Education						
Nil Formal	3.9	(44)	3.0	(15)	4.6	(29)
Grade 1–6	12.8	(146)	13.9	(70)	11.9	(76)
Grade 7–12	37.5	(427)	28.8	(145)	44.3	(282)
Tertiary	7.4	(84)	5.8	(29)	8.6	(55)
Missing data	38.5	(439)	48.5	(244)	30.6	(195)
Maternal height, in cm (n = 781)	157	(6.7)	157	(6.7)	157	(6.7)
Body mass index, in kg/m^2^ (n = 753)	27.0	(5.2)	26.7	(5.0)	27.1	(5.2)
Iron supplementation						
No	5.9	(67)	8.9	(45)	3.4	(22)
Yes	85.2	(971)	71.8	(361)	95.8	(610)
Missing data	8.9	(102)	19.3	(97)	0.8	(5)
Bed net issued during pregnancy						
No	28.7	(327)	41.7	(210)	18.4	(117)
Yes	71.3	(813)	58.3	(293)	81.6	(520)
Birth characteristics						
Mode of delivery						
Vaginal birth	93.6	(1,067)	95.8	(482)	91.8	(585)
Caesarean section	6.4	(73)	4.2	(21)	8.2	(52)
Sex of baby						
Female	48.5	(552)	45.1	(226)	51.3	(326)
Male	51.5	(585)	54.9	(275)	48.7	(310)

Port Moresby General Hospital, Papua New Guinea, 2022

Data are mean (standard deviation) or % (n)

A total of 156 women (13.7%) did not receive any IPTp-SP, 347 women (30.4%) received one dose, and 333 (29.2%) received two doses. Only 304 (26.7%) received the recommended three or more doses of IPTp-SP. Factors associated with receiving < 2 doses of IPTp-SP included lower maternal age (P = 0.014), residing in settlements or a rural location (P < 0.001), having had ≤ 4 antenatal visits (P < 0.001), and no or limited education (P = 0.009) (Table 1). Gravidity, height, and body mass index were not associated with IPTp-SP dose categories. Women who received 2 or more doses of IPTp-SP were more likely to receive iron supplementation (P < 0.001) and a bed net in pregnancy (P < 0.001). Similar associations were noted for other groupings of IPTp-SP dosing (Supplemental Table 3).

A total 65 of 1,110 liveborn babies (5.9%) had LBW, and the overall mean ± SD birth weight was 3275 ± 563 g. Amongst 1,140 women, there were 24 stillbirths (2.1%), 10 early neonatal deaths (0.9%), 34 perinatal deaths (3.0%) and 91 women (8.0%) with the composite outcome LBW and/or perinatal death. Principal factors associated with LBW included young maternal age (P = 0.001), primigravidity (P = 0.004), and having had no or fewer than 4 antenatal visits (P < 0.001). Women of highlander heritage were least likely to have a baby with LBW (P = 0.012) (Supplemental Table 4).

Data on haemoglobin in late pregnancy was available for a subset of women. Women’s mean ± SD haemoglobin was 111 ± 20 g/L and 30.6% (243/793) of women were anaemic, but only 1.4% (11/793) had severe anaemia. There were 14 (1.2%) women who had a documented episode of clinical malaria (confirmed by light microscopy and/or rapid diagnostic test): there was one *P. falciparum* infection, one non-falciparum infection, two mixed infections, and the species was not documented for the remaining 10 infections. These episodes were recorded in six (2.4%) of 249 women with rural or peri-urban residence, and in eight (0.9%) of 891 women with urban residence (P = 0.056). There were no maternal deaths during the study period.

### Impact of intermittent preventive treatment in pregnancy with sulfadoxine-pyrimethamine on birth outcomes

Number of antenatal clinic visits was the only variable that emerged as a confounder of the protective effect of IPTp-SP on LBW (Supplemental Table 1). There was no effect modification of the relationship of IPTp-SP exposure categories with LBW by gravidity or number of antenatal visits. In adjusted analyses using women receiving 0–1 dose of IPTp-SP as the comparator, women receiving 2 or more doses had 50% lower odds of LBW (adjusted odds ratio [aOR] 0.50; 95% confidence interval [CI] 0.26, 0.96), 42% lower odds of preterm birth (aOR 0.58; 95%CI 0.32, 1.04), 51% lower odds of perinatal death (aOR 0.49; 95% CI 0.18, 1.38) and 45% lower odds of the adverse birth outcome composite (aOR 0.55; 95% CI 0.27, 1.12); not all of these associations were statistically significant. Women receiving 2 or more doses of IPTp-SP had babies that weighed on average 108 g (95%CI 31, 186) more compared to women who had 0–1 dose.

There was a dose–response protective effect against LBW with increasing number of IPTp-SP doses (Table 2). Women who received 2 doses versus 0–1 had 45% lower odds of LBW (aOR 0.55, 95%CI 0.27, 1.10), with those receiving ≥ 3 doses having a further 16% (total 61%) reduction (aOR 0.39, 95% CI 0.14, 1.05). There was a corresponding dose–response relationship between mean birth weight and number of IPTp-SP doses received. Compared to women who received 0–1 dose, mean birthweight was 81 g (95% CI −3, 166) and 151 g (95% CI 58, 246) higher amongst women who received 2 doses and ≥ 3 doses of IPTp, respectively. Furthermore, women who received ≥ 3 doses of IPTp-SP compared to 0–1 dose had 67% lower odds of preterm birth (aOR 0.33; 95% CI 0.13, 0.87), but this effect was less pronounced amongst women who only received 2 doses of IPTp-SP (aOR 0.70; 95%CI 0.38, 1.26). There were incremental reductions in the odds of perinatal death and adverse birth outcome with 0–1 dose, 2 doses and ≥ 3 doses (Table 2). Women who received 2 doses versus 0–1 had 43% lower odds of an adverse birth outcome (aOR 0.57, 95% CI 0.32, 1.02) and women who received ≥ 3 doses had 59% lower odds (aOR 0.41, 95% CI 0.19, 0.88).

**Table 2:** Pregnancy outcomes by exposure to 0–1 dose versus 2 doses vs. ≥ 3 doses of sulfadoxine-pyrimethamine

Outcome	N	Number of events or mean	Crude OR or Δ Mean	95% CI	Adjusted OR or Δ Mean^b^	95% CI	P
Low birth weight (< 2,500 g)^a^							
0–1 dose	486	47	Ref		Ref		
2 doses	325	12	0.36	(0.19, 0.69)	0.55	(0.27, 1.10)	0.091
≥ 3 doses	299	6	0.19	(0.08, 0.45)	0.39	(0.14, 1.05)	0.063
Perinatal death^c^							
0–1 dose	503	21	Ref		Ref		
2 doses	333	8	0.57	(0.25, 1.29)	0.54	(0.21, 1.32)	0.173
≥ 3 doses	304	5	0.38	(0.14, 1.02)	0.36	(0.12, 1.08)	0.067
Adverse birth outcome^d^							
0–1 dose	503	61	Ref		Ref		
2 doses	333	19	0.44	(0.25, 0.75)	0.57	(0.32, 1.02)	0.058
≥ 3 doses	304	11	0.27	(0.14, 0.53)	0.41	(0.19, 0.88)	0.022
Preterm birth							
0–1 dose	489	57	Ref		Ref		
2 doses	327	18	0.44	(0.26, 0.77)	0.70	(0.38, 1.26)	0.235
≥ 3 doses	299	6	0.16	(0.07, 0.36)	0.33	(0.13, 0.87)	0.025
Anaemia (< 100 g/L)							
0–1 dose	245	101	Ref				
2 doses	279	84	0.61	(0.43, 0.88)	N/A	N/A	0.008
≥ 3 doses	269	58	0.39	(0.27, 0.58)	N/A	N/A	< 0.001
Birthweight (in grams)							
0–1 dose	486	3146	Ref		Ref		
2 doses	325	3320	174	(97, 252)	81	(−3, 166)	0.059
≥ 3 doses	299	3436	290	(211, 369)	151	(58, 246)	0.002
Haemoglobin (g/L)							
0–1 dose	245	106.4	Ref				
2 doses	279	110.8	4.4	(1.0, 7.9)	N/A	N/A	0.012
≥ 3 doses	269	114.2	7.8	(4.3, 11.3)	N/A	N/A	< 0.001

Port Moresby General Hospital, Papua New Guinea, 2022

^a^Only live births were considered

^b^Adjusted for number of antenatal visits (low birth weight, perinatal death, adverse birth outcome, preterm birth, birthweight)

^c^Considers stillbirths and early neonatal deaths

^d^Defined as a composite of low birth weight and perinatal death

### Impact of differential exposure to intermittent preventive treatment in pregnancy with sulfadoxine-pyrimethamine on anaemia and clinical malaria

There were no measured sociodemographic or clinical factors that emerged as major confounders of the protective effect of IPTp-SP on anaemia (Supplemental Table 2). Women who had received 2 or more doses of IPTp had 50% lower odds of anaemia compared to women who had received 0–1 dose (OR 0.50; 95% CI 0.36, 0.69). There was a dose–response relationship between doses of IPTp and odds of anaemia. Compared to women receiving 0–1 dose, women receiving 2 doses had 39% lower odds, and women receiving ≥ 3 doses had 61% lower odds of anaemia. Corresponding increases in mean haemoglobin were observed with increasing doses of IPTp-SP (Table 2). Clinical malaria in pregnancy was documented amongst 1.6% (8/503) of women receiving 0–1 dose and 1.0% (6/637) of women receiving 2 or more doses (OR 0.59; 95% CI 0.02, 1.71).

### Implementation of malaria control measures

A nested questionnaire-based survey evaluated uptake of malaria prevention measures amongst a subset of women (n = 109) (Table 3). Most respondents received at least one dose of IPTp-SP (93.6%) but only 13.7% had directly observed treatment at clinics. Self-reported side effects were minimal (1.0%). Only one in ten women reported not having a bed net at home, but only one quarter of respondents reported having one net per every two people in their household. Self-reported bed net use was low: only half of women reported using a bed net the night prior to admission, and 45% women reported bed net use prior to conception. Amongst women residing in peri-urban and urban Port Moresby (n = 101), 12 (11.9%) reported travel outside of Port Moresby during the index pregnancy.

**Table 3:** Survey responses regarding geographic mobility, IPTp, and bed net use in pregnancy, Port Moresby General Hospital (n = 109)

Item	Response (%/n)
Travel outside of Port Moresby during pregnancy	
Yes	11.0 (12)^a^
No	81.7 (89)
Not applicable (living in rural area)	7.3 (8)
Years in Port Moresby	
≤ 2 Years	11.9 (12)
> 2 Years	88.1 (89)
Intermittent preventive treatment in pregnancy	
Received sulfadoxine-pyrimethamine	
Yes	93.6 (102)
No	6.4 (7)
If yes, taken under supervision at clinic?	
Yes	13.7 (14)
No	86.3 (88)
If yes, did you take all tablets?	
Every time	94.1 (96)
Sometimes	5.9 (6)
If yes, any side effects	
Yes	1.0 (1)^b^
No	99.0 (101)
Vector control	
Type of house	
Fully screened	67.0 (73)
Partially screened	24.8 (27)
Unscreened	8.2 (9)
Do you have a bed net at home?	
Yes	90.8 (99)
No	9.2 (10)
One net per every two people in household?	
Yes	26.6 (29)
No	73.4 (80)
Used bed net last night?	
Yes	50.5 (55)
No	49.5 (54)
Used bed net during pregnancy?	
Never	41.3 (45)
Sometimes	4.6 (5)
Most of the time	6.4 (7)
All of the time	47.7 (52)
Used bed net since before conception	
Yes	45.0 (49)
No	55.0 (60)

^a^Regions of travel: Southern (8), Highlands (2), Momase (1), unknown (1)

^b^Drowsiness

## Discussion

The present study highlights that receiving two or more doses of IPTp-SP decreased the likelihood of maternal anaemia and LBW delivery amongst pregnant women in Port Moresby, PNG, a low malaria transmission setting. Similarly, women receiving IPTp-SP at least twice had lower odds of perinatal death (not reaching statistical significance). There was a dose–response relationship between birth outcomes and number of IPTp-SP doses, indicating that women receiving more doses of IPTp-SP are better protected. IPTp-SP was well tolerated but only one in ten women had directly observed treatment.

Malaria transmission in PNG is heterogenous, with unstable transmission at higher altitudes, perennial transmission on its north coast, and seasonal transmission in southern provinces [23]. Malaria Indicator Surveys suggest that malaria transmission in the National Capital District is near-absent, whilst transmission in the adjacent Central Province appears to be low [20]. The burden of subpatent malaria infection, which is substantial in other areas of PNG [8], is unknown in this setting. Several symptomatic malaria infections occurred amongst women residing in or near Port Moresby. Some of these malaria episodes may represent *P. vivax* relapses, or new infections acquired during recent travel outside of Port Moresby. Participants who resided in rural/peri-urban areas who subsequently sought care at Port Moresby General Hospital were more likely to be diagnosed with malaria. IPTp-SP and bed nets therefore remain important interventions during pregnancy to prevent and control malaria infection and should be universally deployed. Implementation of IPTp requires strengthening, given only 27% received the recommended three or more doses of IPTp-SP, which was associated with greatest benefit.

IPTp-SP was associated with substantial reductions in adverse pregnancy outcomes in the study setting. Because women in this cohort were not universally screened for parasitaemia, the true burden of infection in the cohort is unknown, as is the proportion of the effect of IPTp-SP on LBW that may have been mediated by preventing/clearing malaria parasitaemia. It is likely that non-malarial mechanisms substantially contributed to better birth outcomes observed amongst women receiving more doses of IPTp-SP in the study setting. Sexually transmitted infections are highly prevalent and a risk factor for LBW, and IPTp-SP was associated with substantial reductions in *Chlamydia trachomatis* and *Neisseria gonorrhoeae* in observational studies and clinical trials in PNG and sub-Saharan Africa [10, 14, 22, 24, 25]. Other mechanisms, such as impacts of SP on the maternal gut and vaginal microbiome, maternal gut function, and other infectious/inflammatory processes, may also be of relevance but require further investigation [13, 15, 16]. Whilst more efficacious anti-malarials may eventually be required for IPTp to counter the possible spread of SP-resistant *P. falciparum* mutants and to provide protection against *P. vivax*, novel IPTp regimens could benefit from retaining SP to maximize protection against LBW [22]. The combination of IPTp-SP plus dihydroartemisinin-piperaquine, which is active against both *P. falciparum* and *P. vivax*, is currently being investigated in Madang Province (NCT05426434).

Antenatal clinics are currently the only platform for the delivery of IPTp-SP in PNG. As expected, infrequent attendance at antenatal clinic was associated with lower number of doses of IPTp-SP and increased risk of LBW. This inverse relationship between number of clinic visits and the incidence of LBW has been observed in other low-resource settings [26, 27]. Not all observational studies exploring the relationship between number of IPTp-SP doses and LBW adjust for number of antenatal visits [28, 29]. This raises the question whether number of doses of IPTp-SP was simply acting as a surrogate for the broader benefits of antenatal clinic attendance. Because antenatal clinic attendance was a principal predictor of LBW in the present cohort, analyses examined whether it was an important confounder of the association between IPTp-SP and LBW. After adjusting for number of antenatal visits in multivariable analyses, the effect of number of IPTp-SP doses on LBW was reduced but persisted. Further statistical analysis excluded effect modification of this relationship by number of antenatal visits and ruled out collinearity between number of antenatal visits and number of SP doses. This suggests that the effect of IPTp-SP on LBW in the study population is genuine and cannot be attributed to more frequent antenatal attendance alone. Furthermore, the present study corroborates findings from a pivotal meta-analysis of the impact of SP dosing on LBW risk, which suggested better protection with three or more doses of SP compared to two or less doses of SP, with similar antenatal clinic coverage in both groups [21].

A reduction in maternal anaemia with increasing number of IPTp-SP doses has been reported previously in higher transmission settings in sub-Saharan Africa [21, 28], but has not been demonstrated in areas of low malaria transmission. Improved haemoglobin levels in women receiving more doses of IPTp-SP in the study setting may reflect SP’s action against asymptomatic malaria infection, the burden of which is uncertain in the study population. Multivariate analysis did not reveal any confounders of the protective effect of IPTp-SP on anaemia, however there may be substantial heterogeneity in how and when haemoglobin was estimated, and the results should be interpreted with caution. The timing of haemoglobin measurement was not readily available during data collection, and as such it remains possible that a significant portion of measurements were taken at the same time or even prior to the first administration of SP. If that was the case, then these findings might reflect women with better baseline haemoglobin levels having better access to IPTp-SP, rather than the impact of SP on anaemia. The collected data did not allow to differentiate between these two possibilities.

The study has several limitations. First, women did not undergo routine screening for malaria infection and only data for clinical malaria was collected, with unknown species profiles for most cases. As the majority of women with parasitaemia are likely to be asymptomatic, and asymptomatic infections are associated with adverse birth outcomes [4], it is unclear how much of a malaria-attributable burden of LBW IPTp-SP may have prevented. Second, the study was a retrospective cohort study, drawing on data documented by health workers in women’s health books, hospital records and the birth register, potentially introducing documentation and recall bias. Notably, LBW was uncommon (5.9%) compared to rural PNG.^14^ At Port Moresby General Hospital, LBW is a triaging criterion for special care nursery admission, which may affect documentation by health workers. Whilst there was heaping at the 2,500 g mark, this did not substantially differ by SP exposure (data not shown). If 37 babies with a birth weight of 2,500 g were recategorized as LBW, the overall prevalence of LBW would be 9.2%. Third, gestational age was determined through a combination of clinical measures such as fundal height, quickening, last menstrual period, and postnatal maturation scoring, all of which are prone to substantial error [30]. As early antenatal attendance and early fetal biometry are rare, pregnancy dating occurs in later gestation, when pregnancies may already be affected by fetal growth restriction, thereby potentially leading to an underestimation of gestational age and a reciprocal overestimation of preterm birth. The association of IPTp-SP dosing and preterm birth should therefore be interpreted with caution. Lastly, residual confounding by hitherto unknown or unmeasured confounders may in part explain some of the associations between IPTp-SP doses and adverse birth outcomes observed in this cohort study.

## Conclusions

Provision of IPTp-SP in a low transmission setting in PNG appears to translate into substantial health benefits, in a dose–response manner. Uptake of ≥ 3 doses of IPTp-SP, which was associated with the greatest protection from LBW and other adverse outcomes, remained low. Unravelling the mechanisms underpinning the possible non-malarial benefits of IPTp-SP may enable clinical evaluation of IPTp-SP in non-malaria endemic settings with a high burden of adverse pregnancy outcomes. Current evidence suggests that IPTp-SP should be universally deployed across different transmission settings within PNG.

### Supplementary Information

Supplementary material 1.

## Data Availability

The data is available upon request from the authors.

## Abbreviations

aOR: Adjusted odds ratio
CI: Confidence interval
HIV: Human immunodeficiency virus
IPTp: Intermittent preventive treatment in pregnancy
IPTp-SP: Intermittent preventive treatment in pregnancy with sulfadoxine-pyrimethamine
LBW: Low birth weight
PNG: Papua New Guinea
OR: Odds ratio
SD: Standard deviation
SP: Sulfadoxine-pyrimethamine
WHO: World Health Organization
