# Supplementary material for: Impact on pregnancy outcomes of intermittent preventive treatment with sulfadoxine-pyrimethamine in urban and peri-urban Papua New Guinea: a retrospective cohort study
Source: Malar J. 2024 Jul 5;23:201. doi: 10.1186/s12936-024-05010-0 (PMC11225125; doi:10.1186/s12936-024-05010-0)
Supplement: Supplementary file 1 — Supplementary material 1. [file 12936_2024_5010_MOESM1_ESM.docx]

**Supplemental Table 1.** Analysis of confounders for low birth weight (n=1,110)

| **Factor** | **Crude odds ratio** | **95% CI** | **P** |  | **Adjusted odds ratio** | **95% CI** | **P** |  | **% Change in odds ratio** | **Missing data** |
| --- | --- | --- | --- | --- | --- | --- | --- | --- | --- | --- |
| Maternal age | 0.26 | (0.11, 0.61) | 0.002 |  | 0.27 | (0.11, 0.63) | 0.002 |  | 2.32 | 0 |
| Primi/Secundigravidae | 0.26 | (0.11, 0.61) | 0.002 |  | 0.26 | (0.11, 0.61) | 0.002 |  | 0.44 | 0 |
| Highland origin | 0.26 | (0.11, 0.61) | 0.002 |  | 0.27 | (0.12, 0.64) | 0.003 |  | 4.09 | 0 |
| Rural/peri-urban residence | 0.26 | (0.11, 0.61) | 0.002 |  | 0.27 | (0.11, 0.63) | 0.003 |  | 3.19 | 0 |
| >=4 Antenatal visits | 0.26 | (0.11, 0.61) | 0.002 |  | 0.53 | (0.21, 1.35) | 0.180 |  | 101.76 | 0 |
| Limited education (<grade 8) | 0.11 | (0.03, 0.48) | 0.003 |  | 0.12 | (0.28, 0.49) | 0.003 |  | 1.11 | 439 |
| Maternal height | 0.23 | (0.07, 0.78) | 0.017 |  | 0.23 | (0.07, 0.78) | 0.017 |  | 0.11 | 359 |
| Body mass index | 0.23 | (0.07, 0.77) | 0.017 |  | 0.22 | (0.07, 0.76) | 0.017 |  | 1.83 | 387 |
| Iron supplementation | 0.34 | (0.14, 0.82) | 0.016 |  | 0.34 | (0.14, 0.82) | 0.016 |  | 0.38 | 102 |
| Bed net issued | 0.26 | (0.11, 0.61) | 0.002 |  | 0.29 | (0.12, 0.67) | 0.004 |  | 9.65 | 0 |
| Female infant | 0.26 | (0.11, 0.61) | 0.002 |  | 0.26 | (0.11, 0.62) | 0.002 |  | 0.20 | 0 |

**Supplemental Table 2.** Analysis of confounders for anaemia in pregnancy (<100 g/L) (n=793)

| **Factor** | **Crude odds ratio** | **95% CI** | **P** |  | **Adjusted odds ratio** | **95% CI** | **P** |  | **% Change in odds ratio** | **Missing data** |
| --- | --- | --- | --- | --- | --- | --- | --- | --- | --- | --- |
| Maternal age | 0.50 | (0.36, 0.71) | <0.001 |  | 0.51 | (0.36, 0.71) | <0.001 |  | 0.43 | 0 |
| Primi/Secundigravidae | 0.50 | (0.36, 0.71) | <0.001 |  | 0.51 | (0.36, 0.71) | <0.001 |  | 0.35 | 0 |
| Highland origin | 0.50 | (0.36, 0.71) | <0.001 |  | 0.51 | (0.36, 0.71) | <0.001 |  | 0.19 | 0 |
| Rural/peri-urban residence | 0.50 | (0.36, 0.71) | <0.001 |  | 0.50 | (0.35, 0.70) | <0.001 |  | 1.66 | 0 |
| >=4 Antenatal visits | 0.50 | (0.36, 0.71) | <0.001 |  | 0.51 | (0.35, 0.73) | <0.001 |  | 0.48 | 0 |
| Limited education (<grade 8) | 0.42 | (0.28, 0.64) | <0.001 |  | 0.42 | (0.28, 0.64) | <0.001 |  | 0.92 | 250 |
| Maternal height | 0.49 | (0.34, 0.71) | <0.001 |  | 0.49 | (0.34, 0.71) | <0.001 |  | 1.78 | 165 |
| Body mass index | 0.49 | (0.33, 0.71) | <0.001 |  | 0.49 | (0.33, 0.71) | <0.001 |  | 1.36 | 181 |
| Iron supplementation | 0.51 | (0.36, 0.71) | <0.001 |  | 0.50 | (0.36, 0.71) | <0.001 |  | 0.72 | 9 |
| Bed net issued | 0.50 | (0.35, 0.71) | <0.001 |  | 0.50 | (0.35, 0.70) | <0.001 |  | 1.17 | 0 |
| Female infant | 0.50 | (0.36, 0.71) | <0.001 |  | 0.51 | (0.36, 0.72) | <0.001 |  | 0.20 | 0 |
| Caesarean section | 0.50 | (0.36, 0.71) | <0.001 |  | 0.51 | (0.36, 0.71) | <0.001 |  | 0.82 | 0 |

**Supplemental Table 3** Characteristics of women at antenatal enrolment, overall and by receipt of sulfadoxine-pyrimethamine (SP). Port Moresby General Hospital, Papua New Guinea, 2022.

|  | | **All Women (n=1,140)** | | **SP 0-2 doses (n=836)** | | **SP ≥3 doses (n=304)** | |
| --- | --- | --- | --- | --- | --- | --- | --- |
| Age, in years | | 26.6 | (6.0) | 26.3 | (6.1) | 27.2 | (5.7) |
| Gravidity | |  |  |  |  |  |  |
|  | Primigravid | 38.5 | (439) | 39.4 | (329) | 36.2 | (110) |
|  | Secundigravid | 28.4 | (324) | 27.9 | (233) | 29.9 | (91) |
|  | Multigravid | 33.1 | (377) | 32.8 | (274) | 33.9 | (103) |
| Region of Origin | |  |  |  |  |  |  |
|  | Southern | 47.5 | (542) | 49.8 | (416) | 41.5 | (126) |
|  | Highlands | 40.4 | (461) | 38.8 | (324) | 45.1 | (137) |
|  | Islands | 4.7 | (53) | 4.2 | (35) | 5.9 | (18) |
|  | Momase | 7.4 | (84) | 7.3 | (61) | 7.6 | (23) |
| Place of Residence | |  |  |  |  |  |  |
|  | Urban | 47.9 | (546) | 43.2 | (361) | 60.9 | (185) |
|  | Settlement | 30.3 | (345) | 33.0 | (276) | 22.7 | (69) |
|  | Peri-urban/Rural | 21.8 | (249) | 23.8 | (199) | 16.4 | (50) |
| Antenatal clinic booking | |  |  |  |  |  |  |
|  | No | 7.8 | (89) | 10.6 | (89) | 0.0 | (0) |
|  | Yes | 92.2 | (1,051) | 89.4 | (747) | 100.0 | (304) |
| Number of antenatal clinic visits | |  |  |  |  |  |  |
|  | None | 7.8 | (89) | 10.7 | (89) | 0.0 | (0) |
|  | 1-3 | 31.0 | (353) | 40.9 | (342) | 3.6 | (11) |
|  | 4-7 | 44.7 | (509) | 39.6 | (331) | 58.6 | (178) |
|  | 8+ | 16.6 | (189) | 8.8 | (74) | 37.8 | (115) |
| Education | |  |  |  |  |  |  |
|  | Nil Formal | 3.9 | (44) | 3.7 | (31) | 4.3 | (13) |
|  | Grade 1-6 | 12.8 | (146) | 13.5 | (113) | 10.9 | (33) |
|  | Grade 7-12 | 37.5 | (427) | 34.6 | (289) | 45.4 | (138) |
|  | Tertiary | 7.4 | (84) | 6.2 | (52) | 10.5 | (32) |
|  | Missing data | 38.5 | (439) | 42.0 | (351) | 28.9 | (88) |
| Maternal height, in cm (n=781) | | 157 | (6.7) | 157 | (6.6) | 158 | (6.7) |
| Body mass index, in kg/m^2^ (n=753) | | 27.0 | (5.2) | 26.9 | (5.0) | 27.0 | (5.5) |
| Iron supplementation | |  |  |  |  |  |  |
|  | No | 5.9 | (67) | 6.7 | (56) | 3.6 | (11) |
|  | Yes | 85.2 | (971) | 81.5 | (681) | 95.4 | (290) |
|  | Missing data | 8.9 | (102) | 11.8 | (99) | 1.0 | (3) |
| Bed net issued during pregnancy | |  |  |  |  |  |  |
|  | No | 28.7 | (327) | 32.4 | (271) | 18.4 | (56) |
|  | Yes | 71.3 | (813) | 67.6 | (565) | 81.6 | (248) |
| **Birth characteristics** | | | | | | | |
| Mode of delivery | |  |  |  |  |  |  |
|  | Vaginal birth | 93.6 | (1,067) | 94.6 | (791) | 90.8 | (276) |
|  | Caesarean section | 6.4 | (73) | 5.4 | (45) | 9.2 | (28) |
| Sex of baby | |  |  |  |  |  |  |
|  | Female | 48.5 | (552) | 47.5 | (396) | 51.3 | (156) |
|  | Male | 51.5 | (585) | 52.5 | (437) | 48.7 | (148) |

Data are mean (standard deviation) or % (n).

**Supplemental Table 4.** Association of maternal and neonatal characteristics with low birth weight (LBW, <2,500 g) on crude analysis,

Port Moresby General Hospital (n=1,110)

| **Characteristic** | |  | | | |
| --- | --- | --- | --- | --- | --- |
|  | | **% LBW** | **(n with LBW/**  **total)** | **Odds**  **(95% CI)** | ***P*** |
| **Maternal age** | |  |  |  |  |
|  | >20 years | 4.8 | (44/919) | Ref |  |
|  | ≤20 years | 11.0 | (21/191) | 2.46 (1.42, 4.24) | 0.001 |
| **Gravidity** | |  |  |  |  |
|  | Primigravidae | 8.6 | (37/430) | Ref |  |
|  | Secundigravidae | 4.8 | (15/313) | 0.50 (0.29, 0.99) | 0.047 |
|  | Multigravidae | 3.5 | (13/367) | 0.39 (0.20, 0.75) | 0.004 |
| **Region of origin** | |  |  |  |  |
|  | Southern | 7.0 | (37/526) | Ref |  |
|  | Highlands | 3.3 | (15/450) | 0.46 (0.25, 0.84) | 0.012 |
|  | Islands | 15.4 | (8/52) | 2.40 (1.05, 5.48) | 0.037 |
|  | Momase | 6.1 | (5/82) | 0.86 (0.33, 2.25) | 0.756 |
| **Residence** | |  |  |  |  |
|  | Urban | 5.3 | (28/530) | Ref |  |
|  | Urban settlement | 5.0 | (17/337) | 0.95 (0.51, 1.77) | 0.877 |
|  | Peri-urban/rural | 8.2 | (20/243) | 1.61 (0.89, 2.92) | 0.118 |
| **Number of antenatal visits** | |  |  |  |  |
|  | 0-3 visits | 10.8 | (46/427) | Ref |  |
|  | ≥4 visits | 2.8 | (19/683) | 0.24 (0.14, 0.41) | <0.001 |
| **Educational status** | |  |  |  |  |
|  | ≤Grade 7 | 5.2 | (26/500) | Ref |  |
|  | Grade 8 to tertiary | 6.5 | (12/184) | 1.27 (0.63, 2.58) | 0.504 |
| **Maternal height, cm** | |  |  |  |  |
|  | ≤150 | 5.0 | (6/119) | Ref |  |
|  | >150 | 3.7 | (24/644) | 0.73 (0.29, 1.82) | 0.499 |
| **Body mass index (kg/m^2^) at booking** | |  |  |  |  |
|  | <25^1^ | 6.3 | (18/287) | Ref |  |
|  | 25 to <30 | 2.9 | (8/276) | 0.45 (0.19, 1.04) | 0.063 |
|  | ≥ 30 | 2.3 | (4/173) | 0.35 (0.12, 1.06) | 0.064 |
| **Iron supplementation during pregnancy** | |  |  |  |  |
|  | No | 4.6 | (3/65) | Ref |  |
|  | Yes | 4.6 | (44/953) | 1.00 (0.30, 3.31) | 1.000 |
| **ITN issued** | |  |  |  |  |
|  | No | 9.3 | (29/312) | Ref |  |
|  | Yes | 4.5 | (36/798) | 0.46 (0.28, 0.77) | 0.003 |
| **Sex of baby** | |  |  |  |  |
|  | Male | 5.4 | (31/570) | Ref |  |
|  | Female | 6.1 | (33/537) | 1.14 (0.69, 1.89) | 0.615 |

^1^Includes 15 women with a body mass index <18.5
